# Sub-acute Toxicity in Non-cancerous Tissue and Immune-Related Adverse Events of a Novel Combination Therapy for Cancer

**DOI:** 10.3389/fonc.2019.01504

**Published:** 2020-01-14

**Authors:** Kelly J. McKelvey, Amanda L. Hudson, Ramyashree Prasanna Kumar, Thomas Eade, Stephen J. Clarke, Helen R. Wheeler, Connie I. Diakos, Viive M. Howell

**Affiliations:** ^1^Bill Walsh Translational Cancer Research Laboratory, Kolling Institute, The University of Sydney Northern Clinical School and Northern Sydney Local Health District, St Leonards, NSW, Australia; ^2^Sydney Vital Translational Cancer Research Centre, Royal North Shore Hospital, St Leonards, NSW, Australia; ^3^The Brain Cancer Group, St Leonards, NSW, Australia; ^4^Northern Sydney Cancer Centre, Royal North Shore Hospital, St Leonards, NSW, Australia

**Keywords:** cancer, radiation, immunotherapy, inflammation, toxicity

## Abstract

Brain, lung, and colon tissue experience deleterious immune-related adverse events when immune-oncological agents or radiation are administered. However, there is a paucity of information regarding whether the addition of radiation to immuno-oncological regimens exacerbates the tissue inflammatory response. We used a murine model to evaluate sub-acute tissue damage and the systemic immune response in C57Bl/6 mice when administered systemic anti-programmed cell death protein 1 (αPD-1) immunotherapy alone or in combination with stereotactic fractionated 10 gray/5 X-ray radiation to normal brain, lung or colon tissue. The model indicated that combinatorial αPD-1 immunotherapy and radiation may alter normal colon cell proliferation and cerebral blood vasculature, and induce systemic thrombocytopenia, lymphopenia, immune suppression, and altered immune repertoire (including interleukin-1β). Therein our data supports close monitoring of hematological and immune-related adverse events in patients receiving combination therapy.

## Introduction

While immunotherapies have the potential to revolutionize therapy there is limited understanding of their interaction with radiation in healthy tissues. To date a number of factors have restricted the assessment of treatment efficacy of check point inhibitors in combination with radiation in cancer patients. These include treatment discontinuation in ~10% of patients due to immune-related adverse events and unacceptable level of injury to healthy tissue (1). These factors sometimes stem from the complex immunostimulation arising from the combination of radiation and chemotherapy in these patients. As such it is not clear if patients may derive greater long-term benefit from combined use of radiotherapy (RT) and an immunotherapy checkpoint inhibitor.

Evidence demonstrating safety, i.e., minimal tissue damage and immune-related adverse events in normal/healthy tissue is lacking as it is unethical to administer RT to healthy tissue in people. Immunotherapy alone is reported to induce a range of side effects most commonly in skin, gastrointestinal tract, lung, and endocrine glands. While the majority of immune-related adverse events are mild to moderate, serious and life threatening events have been reported (2). These led to the introduction of consensus recommendations from the Society for Immunotherapy of Cancer Toxicity Management Working Group (3), and the establishment of clinical practice guidelines for the management of toxicities from immunotherapy by the European and American Medical Societies (4, 5). A systematic review and meta-analysis of 13 studies of patients receiving the anti-programmed cell death protein 1 (αPD-1)/PD-L1 immunotherapies nivolumab, pembrolizumab, or atezolizumab—in combination with chemotherapy—identified increased odds ratios for the incidence of immune associated toxicities hypothyroidism, pneumonitis, colitis, hypophysitis (6), and acute interstitial nephritis (7). The immune-related adverse events associated with checkpoint inhibitors are thought to be linked to immunostimulation and reprogramming of the immune system, leading to a loss of immune tolerance (7). Such adverse events may be exacerbated by RT, where there is a rising paradigm of an immunostimulatory effect of RT in patients undergoing treatment with immune checkpoint inhibitors. Furthermore, the various checkpoint inhibitors differentially modulate T-cell responses leading to distinct toxicity patterns, kinetics, and dose–toxicity relationships. These need to be better understood before widely utilizing combinations of RT and immunotherapy in the clinical setting.

Radiation activates an interconnected network of inflammatory and immune response pathways inducing a host of changes to the tissue microenvironment (8). Lung and colon tissues display two of the most common immune-related adverse events in pneumonitis and colitis, while adverse events in brain tissue, such as encephalitis and neuropathy, are relatively rare (2–5). Due to the idiosyncratic nature of adverse events affected the brain, lung and colon tissues, we sought to pre-clinically model the subacute response to potentially predict future immune-related adverse events.

To understand whether the addition of RT to immuno-oncology agents exacerbates the immune response in normal brain lung and colon tissues, compared to immune-oncology agents alone, we used a murine model to characterize and quantify the sub-acute (day 28) tissue damage and local and systemic immune responses following combined fractionated stereotactic RT and αPD-1 immunotherapy. We hypothesized that this would identify systemic immune markers that could identify immune-mediated adverse events in brain, lung and colon tissues.

## Materials and Methods

### Mice

The study was reviewed and approved by the Northern Sydney Local Heath District Animal Ethics Committee, Royal North Shore Hospital, St. Leonards, Australia (Approval #RESP/17/205). Eight week old male C57Bl/6.Kearn's mice were kept on 12 h day/night light cycles with standard chow and water provided *ad libitum*. Mice were randomly allocated into 6 mice per treatment group and monitored for well-being by trained animal house staff prior to being humanely killed by cardiac puncture under anesthesia at the pre-determined endpoint of 28 days. C57Bl/6 mice were used as this is the background strain to commonly used syngeneic cancer models.

### Immunotherapy

Mice were treated with *InVivoMab* rat anti-mouse PD-1 (RMP1-14; 200 μg/dose; BE0146; BioXCell) or rat IgG2a isotype control, anti-trinitrophenol (2A3; 200 μg/dose; BE0089; BioXCell) in 100 mirolitres (μl) PBS by intraperitoneal injection every 3 days for 5 doses (day 8, 11, 14, 17, 20) alone or in combination with fractionated stereotactic RT.

### Fractionated Stereotactic Radiotherapy

Cone beam computed tomography (CBCT)-guided stereotactic radiation was delivered to the brain (right hemisphere), lung (right) or colon (sigmoid colon) region at 10 Gray (Gy)/5 X-ray on days 1, 2, 3, 4, 5 using the Small Animal Radiation Research Platform (SARRP; Xstrahl Inc.), 5 × 5 millimeter (mm) collimator, 220 kV, 13 mA, 0.15 mm copper filter, 3.71 gray (Gy)/minute (min), 360° Arc (–180 to 180°) alone or in combination with immunotherapy. Dose output and half-value layer were verified by 0.6 cm^3^ Waterproof Farmer^®^ Chamber (PTW TN30013; −400 V) under reference conditions; 35 cm source to axis distance, 2 centimeter (cm) solid-state depth.

An additional 4 centigray (cGy) was delivered to each animal during CBCT imaging dose − 60 kV, 0.8 mA, 360 projections, fine focus as determined by MOSFET dosimetry MOSkin developed by the Center for Medical Radiation Physics of the University of Wollongong, Australia (9, 10) positioned in the center of a 3D printed modular CBCT cylindrical phantom (mass density ρ = 1.17 g/cm^3^) (11).

To estimate the radiation dose delivered to the targeted tissue region and non-targeted organs at risk, the SARRP Dose Volume Histogram (DVH) in the Treatment Planning Software (MuriPlan^®^ Xstrahl Inc.) was utilized. Tissues were contoured using the acquired CBCT images and Digimouse murine anatomy atlas (available at: https://neuroimage.usc.edu/neuro/Digimouse) (12, 13) (Supplementary Figure 1). Following application of the planned treatment beam, data indicated the mean dose per fraction delivered to the targeted brain region was 199.11 cGy at a volume of 0.02 cubic centimeters (cc), colon 169.57 cGy at 0.06 cc, and lung 158.39 cGy at 0.01 cc. Doses to non-targeted organs at risk were highest in tissues surrounding the brain—mean 84.88 cGy, anorectal region−60.95 cGy, and tissues surrounding the right lung−26.57 cGy (Supplementary Table I).

### Histopathology

Brains were harvested and fixed in 10% v/v neutral buffered formalin for 24 h before embedding in paraffin wax. Four micrometer (4 μm) sections were rehydrated and microwave antigen retrieval performed in citrate buffer, pH 6.0. Next, sections were incubated with 2.5% v/v normal goat serum, followed by primary antibody for 1 h at room temperature. Primary antibodies were Ki67 (0.08 μg/ml; 12202; Cell Signaling Technologies), CD31 (0.013 μg/ml; 77699; Cell Signaling Technologies) and γ-H2AX (0.06 μg/ml; ab11174; Abcam). Finally, sections were incubated with ImmPRESS™ HRP goat anti-rabbit IgG polymer (MP-7451; Vector Labs) for 30 min at room temperature and detected with NovaRed (SK-48000; VectorLabs).

Slides were scanned using the Aperio AT2 Digital Pathology Scanner and five digital images per section at 20x magnification captured using Aperio ImageScope (v12.3.2.8013; Leica Biosytems). Ki67 and γ-H2AX positive staining was quantified by ImmunoRatio ImageJ plugin (v1.0c, 14.2.2011; http://jvsmicroscope.uta.fi/immunoratio/). CD31 positive vessels were enumerated and measured using the Microvessel-Segmentation MATLAB plugin (14).

### Hematology and Flow Cytometry

One milliliter (ml) of whole blood was collected via cardiac puncture into K_3_EDTA tubes (Minicollect^®^ Greiner Bio-One) and assessed by a COULTER^®^ Ac-T diff hematology analyzer with Vet App 1.06 (Beckman Coulter).

Using 100 μl whole blood, 1 × 10^6^ splenocytes and 1 × 10^6^ bone marrow-derived cells and red blood cells were lysed and leukocytes stained with a cocktail of antibodies—volume denoted per test; CD25-BV421 (1 μl; 564370), FV510-BV510 (1 μl; 564406), CD80-BV605 (1 μl; 563052), NK1.1-BV650 (1 μl; 564143), CD4-BV711 (0.25 μl; 563726), CD117-BV786 (1 μl; 564012), CD11b-BB515 (0.25 μl; 564454), CD19-PerCP/Cy5.5 (1 μl; 551001), CD115-PE (0.25 μl; 565249), Ly6G-PE/CF594 (0.06 μl; 562700), CD3-PE/Cy7 (1 μl; 552774), CD206-AF647 (1 μl; 565250), CD8a-AF700 (0.25 μl; 557959), Ly6C-APC/Cy7 (0.5 μl; 560596; all BD Biosciences). Acquired using a BD LSRFortessa™ and analyzed using BD FACSDiva™ Software version 6 (BD Biosciences).

Immune cell populations were defined as CD3^+^ T cell, CD3^+^CD4^+^ helper T cell (Th), CD3^+^CD4^+^CD25^+^ regulatory T cell (Treg), CD3^+^CD8 cytotoxic T cell (Tc), CD3^−^NK1.1^+^ natural killer (NK) cells, CD3^+^NK1.1^+^ (NK/T), CD115^+^CD11b^+^ monocytes (Mono), CD115^+^CD11b^+^CD80^+^ macrophage type 1 (M1), CD115^+^CD11b^+^CD206^+^ macrophage type 2 (M2), CD115^−^CD11b^+^ dendritic cells (DC), CD115^−^CD11b^+^Ly6C^high^Ly6G^−^ monocytic-myeloid derived suppressor cells (M-MDSC), CD115^−^CD11b^+^Ly6C^low^Ly6G^high^ polymorphonuclear-myeloid derived suppressor cells (PMN-MDSC), CD117^+^ hematopoietic stem cell (HSC), CD19^+^ B cells and expressed as a percentage of the parent population.

### Multiplex Immunoassays

Plasma was obtained by centrifugation of whole blood (500 × g, 5 min at room temperature). Mouse cytokine 23-plex immunoassay (Bio-Plex^®^ Bio-Rad Laboratories) and chromogenic sandwich enzyme-linked immunosorbent assay (ELISA) for transforming growth factor (TGF)-β1 (DY1679; R&D Systems) were performed as per the manufacturer's instructions.

### Statistical Analyses

Animal weight between treatment groups was assessed by Two Way Repeated Measures Analysis of Variance (ANOVA) with Tukey's Multiple Comparison Test. Normality of the data was confirmed by the D'Agostino-Pearson omnibus test. Histological data are expressed as the mean of 5 high power fields ± standard error of the mean (SEM). Hematology, flow cytometry and chemokine/cytokine data are expressed as mean ± standard deviation (SD). Two Way Analysis of Variance (ANOVA) with Tukey's Multiple Comparison Test were performed to compare treatments groups for each cell phenotype or cytokine using Prism 7 for Windows (GraphPad Software, Inc.).

## Results

### No Change in Animal Weight

Animal weights were not significantly altered by αPD-1 immunotherapy or RT of the brain, colon or right lung regions [*F*_(8, 45)_ = 1.347; *p* = 0.25; Supplementary Figure 2]. No animals demonstrated signs of poor body condition up to day 28; there was no skin irritation, hair loss, diarrhea or labored breathing. As expected, animal weight significantly increased with time [*F*_(9, 405)_ = 292.5; *p* < 0.0001].

### Reduced Ki67^+^ Proliferation and Blood Vasculature Following Combination Therapy

**Figure 1:**
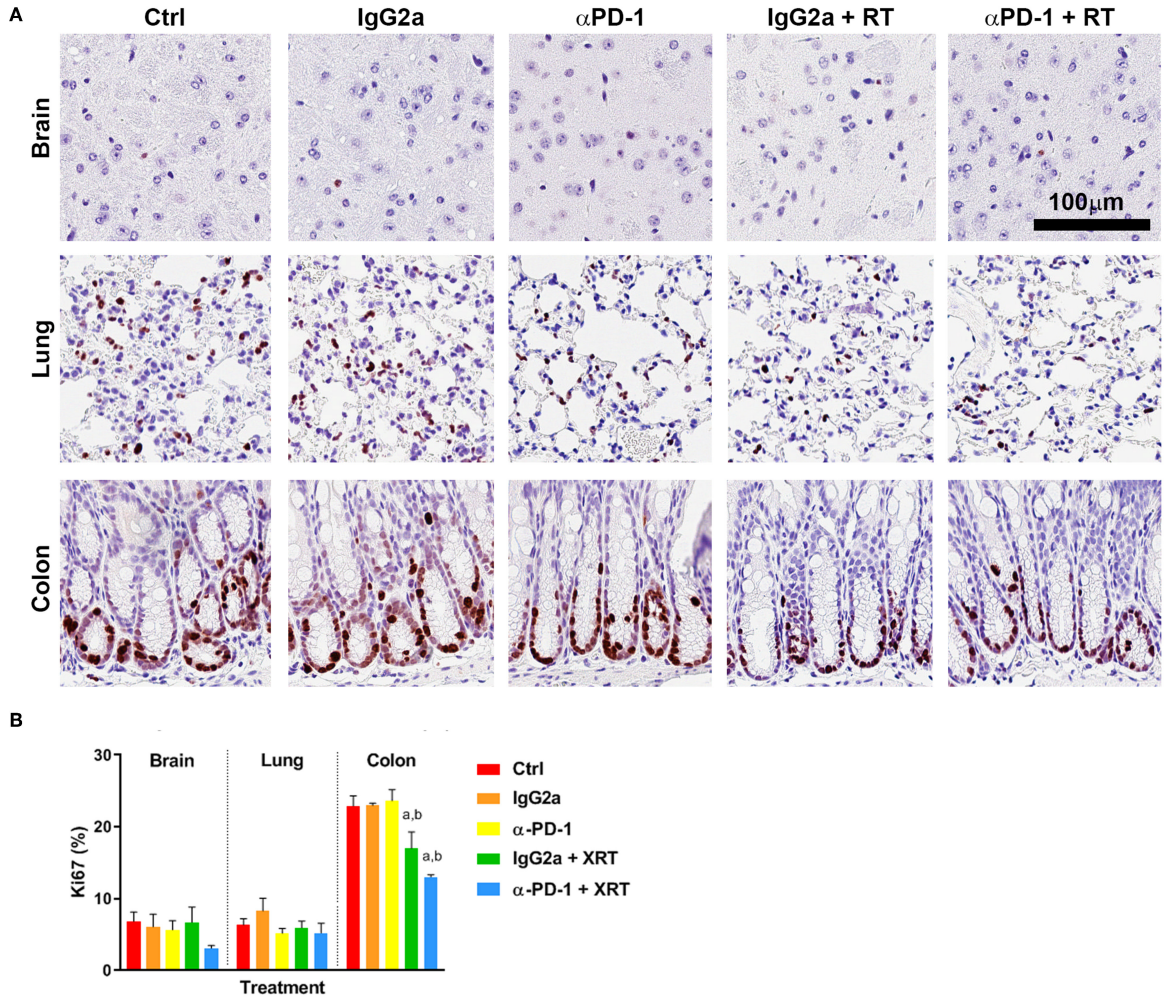
Ki67^+^ staining at the irradiation sites of murine brain, lung and colon tissue **(A)**. Scale bar, 100 μm. Quantitation of five high power fields for each murine tissue (*N* = 6 per time point; **B**). Data are expressed as mean ± SEM per high power field. ^a^*p* < 0.05 vs. Ctrl, ^b^*p* < 0.05 vs. IgG2a by Tukey's multiple comparison test.

Quantification of Ki67^+^ staining showed low levels of proliferation in normal brain glial cells and lung stromal cells, and high proliferation in the actively regenerating colon progenitor cells at the base of the intestinal crypts (Figure 1A). Combined RT and αPD-1 decreased Ki67^+^ 45% in brain (*p* = 0.09; Figure 1) and 25% in colon tissue (*p* = 0.0003; Figure 1) compared to αPD-1 alone. Data indicate that normal brain and colon tissue is susceptible to radiation-induced changes in cellular proliferation.

**Figure 2:**
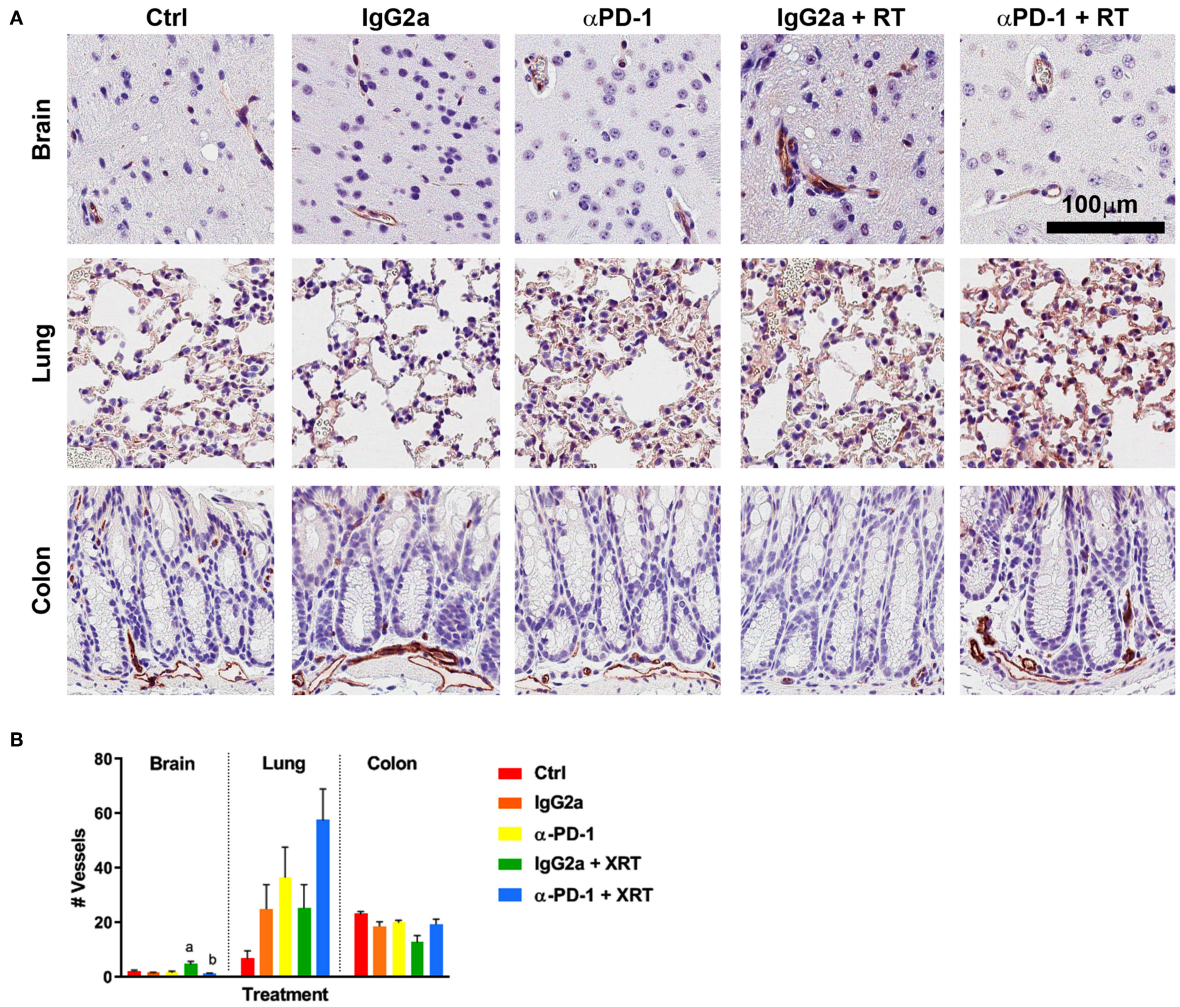
CD31^+^ staining at the irradiation sites of murine brain, lung and colon tissue **(A)**. Scale bar, 100 μm. Quantitation of five high power fields for each murine tissue (*N* = 6 per time point; **B**). Data are expressed as mean ± SEM per high power field. ^a^*p* < 0.05 vs. IgGa, ^b^*p* < 0.05 vs. IgG2a + RT by Tukey's multiple comparison test.

To determine whether the combination therapy of fractionated stereotactic radiation and αPD-1 immunotherapy would impact blood vasculature, CD31^+^ blood vessels were quantified at the targeted tissue region. In brain tissue, combined RT + αPD-1 reduced blood vasculature 3-fold (*p* = 0.001; Figure 2), while in lung tissue combined treatment increased blood vasculature 126% (*p* = 0.06; Figure 2) compared to RT + IgG2a. Other vasculature parameters assessed were vessel thickness, perimeter, area, luminal area, and vessel eccentricity—but did not differ significantly between the treatment groups (data not shown). Data show that RT + αPD-1 immunotherapy augments blood vasculature in normal brain tissue.

**Figure 3:**
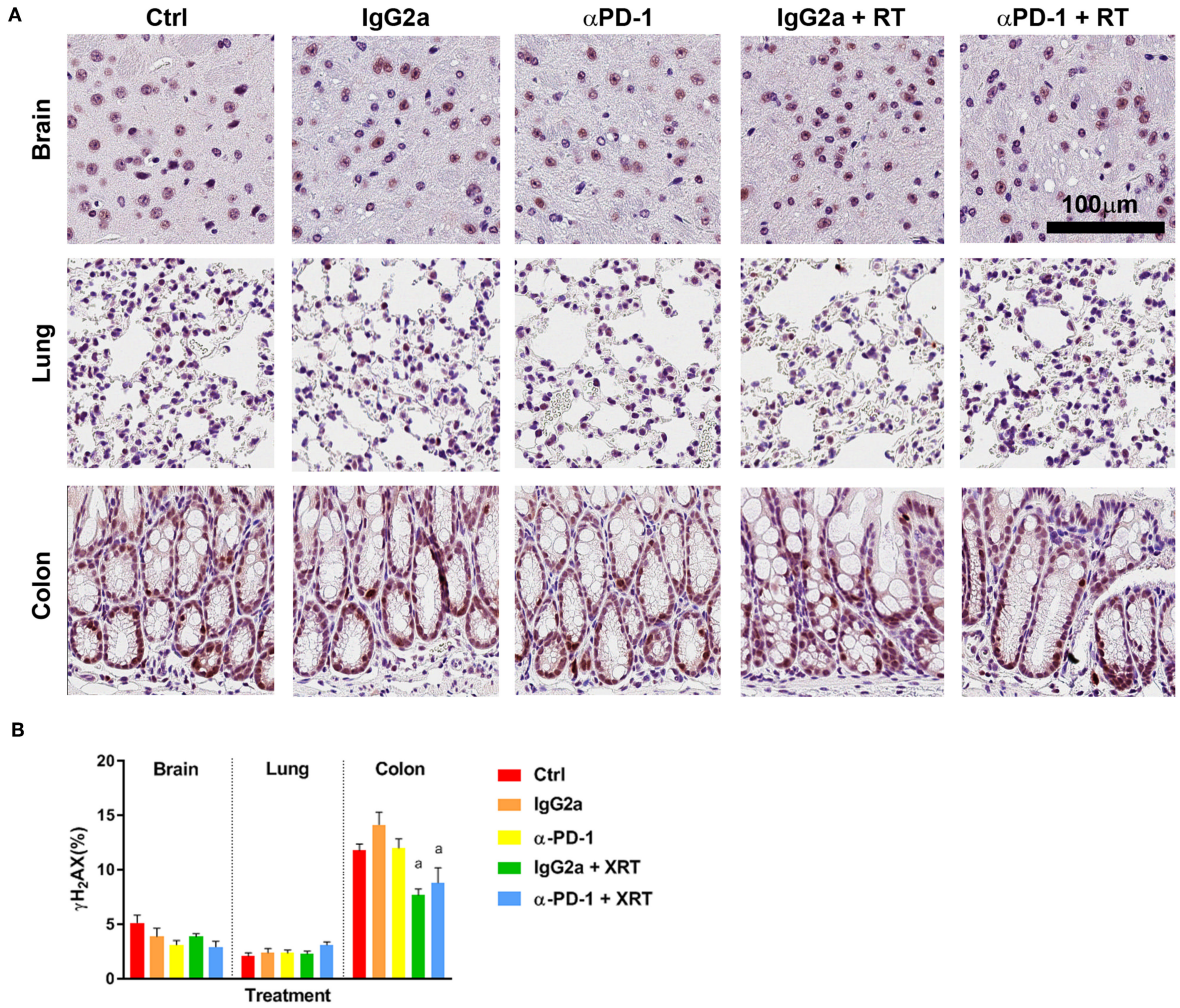
γ-H2AX^+^ staining at the irradiation sites of murine brain, lung and colon tissue **(A)**. Scale bar, 100 μm. Quantitation of five high power fields for each murine tissue (*N* = 6 per time point; **B**). Data are expressed as mean ± SEM per high power field. ^a^*p* < 0.05 vs. Ctrl by Tukey's multiple comparison test.

To determine whether RT-induced damage was prolonged at the sites of irradiation when αPD-1 immunotherapy is combined, γ-H2AX staining was performed to identify double stranded DNA breaks marked for repair (15). As expected, baseline γ-H2AX^+^ staining was higher in colon tissue than brain and lung due to more rapid cell regeneration. Somewhat contradictory, γ-H2AX^+^ staining was significantly reduced by 50% in irradiated colon tissue irrespective of αPD-1 immunotherapy (Figure 3). Data show that there is exacerbation of persistent radiation-induced DNA damage following combined radiation and αPD-1 treatment.

**Figure 4:**
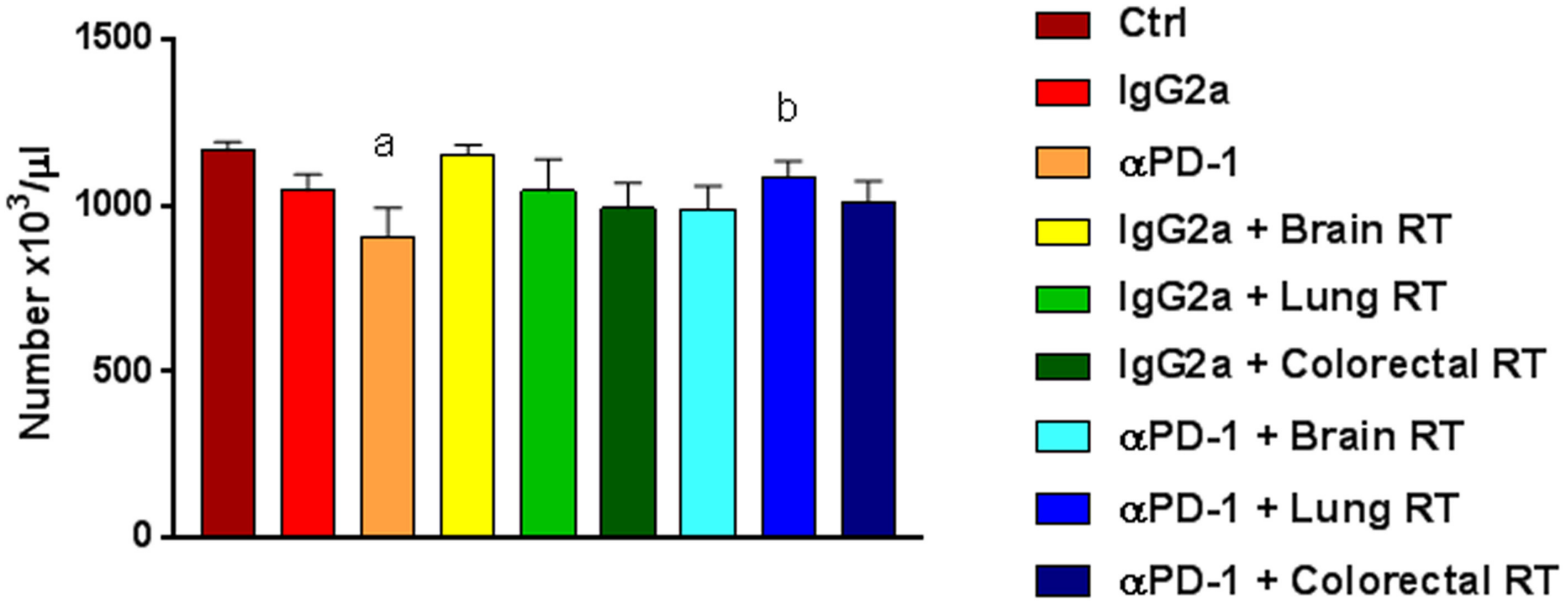
Platelets in mice treated with αPD-1 and/or 10Gy/5 X-ray irradiation of brain, colon or right lung region. Blood was harvested at day 28 and quantitated by multi-color flow cytometry. Data are expressed as mean ± SD absolute count of platelets (*N* = 6 mice per treatment group). One Way ANOVA ^a^*p* < 0.05 vs. Ctrl; ^b^*p* < 0.05 vs. αPD-1 by Tukey's Multiple Comparison Test.

Combination therapy alters immune cell populations in systemic compartments. To assess the systemic immune response to combined stereotactic radiation and αPD-1 immunotherapy, hematological parameters and immune cell populations in the spleen, bone marrow and peripheral blood were quantified by flow cytometric analysis. Of the hematological parameters assessed, αPD-1 suppressed platelet numbers when compared to control (905.5 ± 86.8 vs. 1164.4 ± 26.6, *p* = 0.0006) but normalized in animals that received irradiation of the lung tissue (1087.3 ± 45.9 vs. 905.5 ± 86.8, *p* = 0.034; Figure 4).

**Figure 5:**
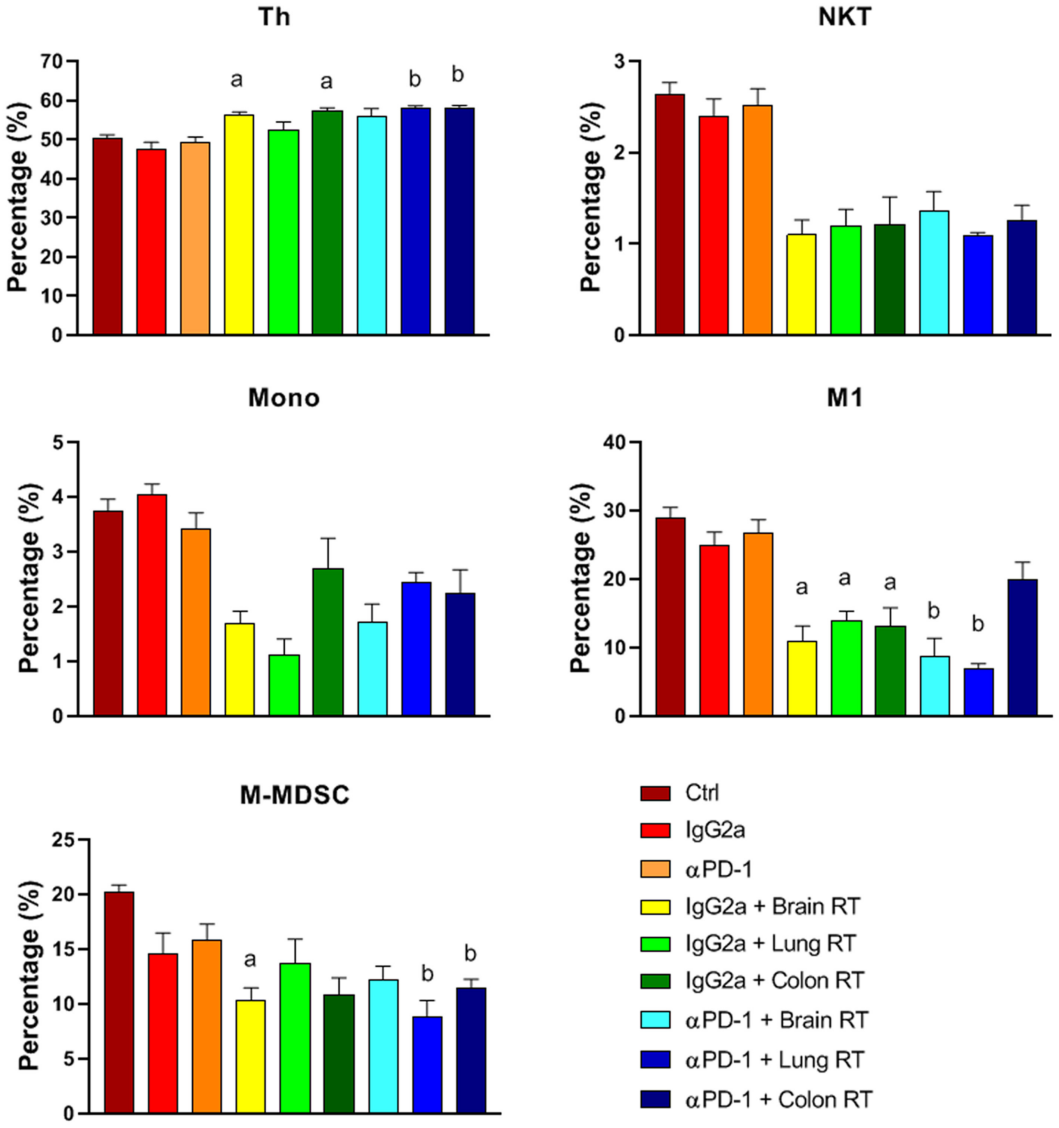
Splenic immune cell populations from mice treated with αPD-1 and/or 10 Gy/5 X-ray irradiation of brain, lung and colon tissue. Cells were harvested at day 28 and quantitated by multi-color flow cytometry. Data are expressed as mean ± SD percentage of parent population (%; *N* = 6 mice per treatment group). Two Way ANOVA ^a^*p* < 0.05 vs. IgG2a; ^b^*p* < 0.05 vs. αPD-1 by Tukey's Multiple Comparison Test.

Splenic CD4^+^ helper T (Th) cells increased 20%, while M-MDSC and M1 decreased 40–80% following combined treatment compared to αPD-1 alone (*p* < 0.05; Figure 5). In addition, splenic NK/T and monocytes were suppressed following irradiation irrespective of αPD-1 immunotherapy, though these did not reach significance.

**Figure 6:**
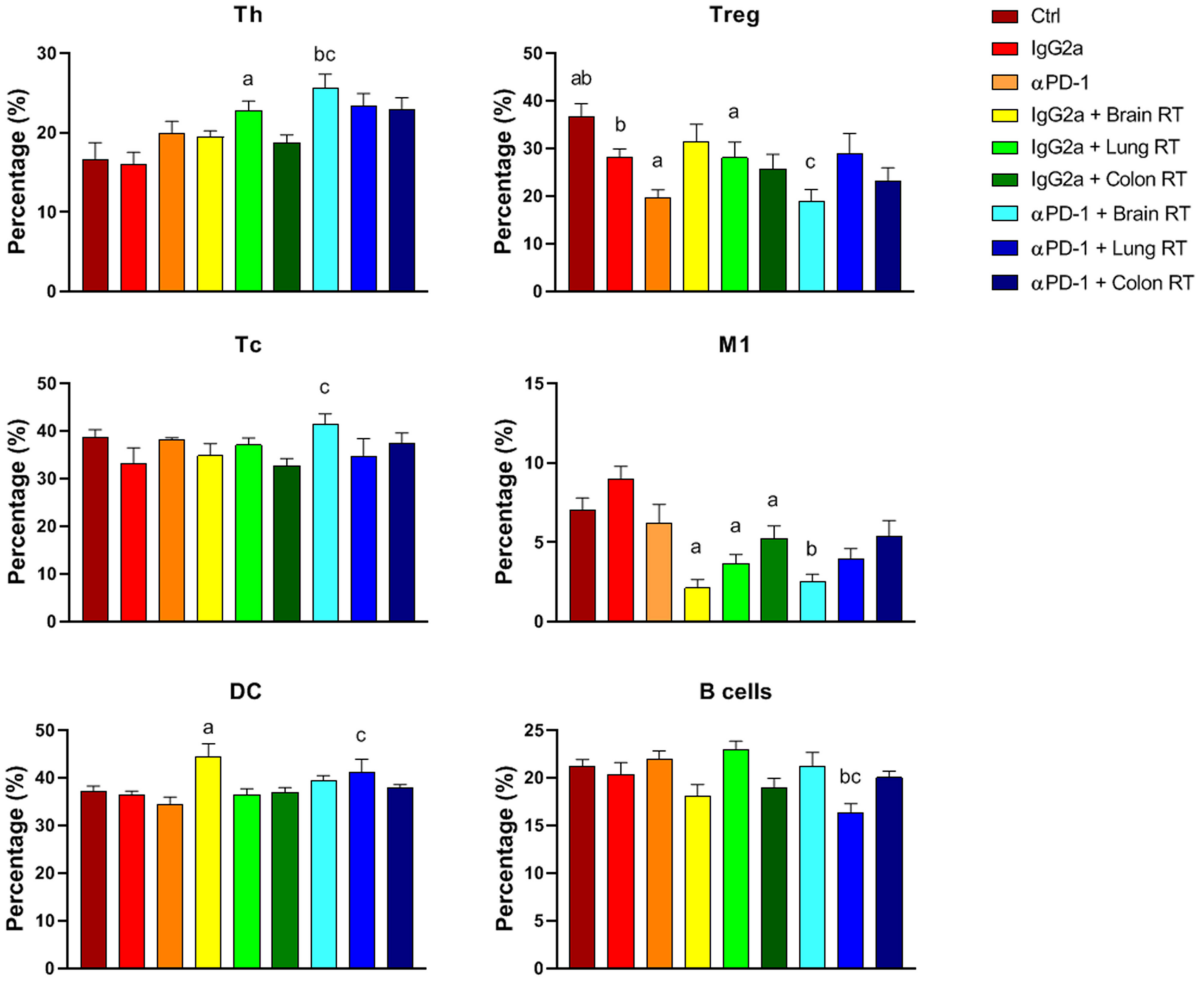
Bone marrow immune cell populations from mice treated with αPD-1 and/or 10 Gy/5 X-ray irradiation of brain, lung, and colon tissue. Cells were harvested at day 28 and quantitated by multi-color flow cytometry. Data are expressed as mean ± SD percentage of parent population (%; *N* = 6 mice per treatment group). Two Way ANOVA ^a^*p* < 0.05 vs. IgG2a; ^b^*p* < 0.05 vs. αPD-1; ^c^*p* < 0.05 vs IgG2a + RT by Tukey's Multiple Comparison Test.

In bone marrow, the most striking finding was the reduction in M1 macrophages with RT independent of immunotherapy (*p* < 0.05; Figure 6). These reductions closely mirrored the responses of splenic M1 macrophages (Figure 5). Furthermore, in bone marrow Th, Tc and DCs were increased while Tregs and B cells decreased following brain or lung RT + αPD-1 compared to monotherapies (*p* < 0.05; Figure 6). Data suggests that the addition of RT to αPD-1 monotherapy may enhance an immune response with increased Th, Tc and DCs. Additionally, a reduction of bone marrow Tregs following combination therapy in lung, may contribute to lymphopenia or immune suppression due to the role of Tregs in B cell differentiation for HSCs (16). Notably, the proportion of HSCs was not altered by treatment (Supplementary Figures 3–5).

**Figure 7:**
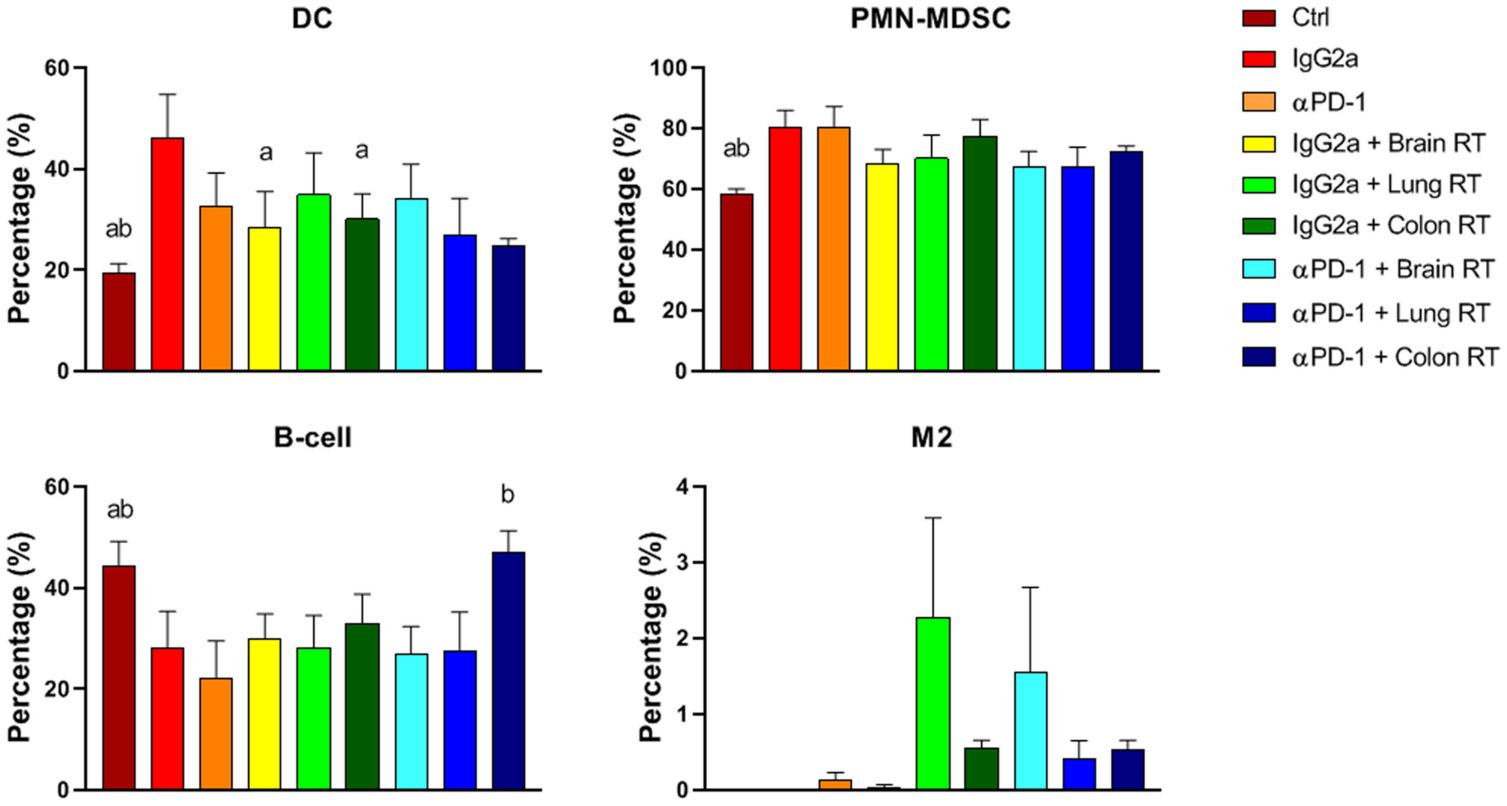
Peripheral blood immune cell populations from mice treated with αPD-1 and/or 10 Gy/5 X-ray irradiation of brain, lung and colon tissue. Cells were harvested at day 28 and quantitated by multi-color flow cytometry. Data are expressed as mean ± SD percentage of parent population (%; *N* = 6 mice per treatment group). Two Way ANOVA ^a^*p* < 0.05 vs. IgG2a; ^b^*p* < 0.05 vs. αPD-1 by Tukey's Multiple Comparison Test.

In peripheral blood, colon RT + αPD-1 increased B-cells 2.1-fold compared to αPD-1 alone (*p* < 0.0001; Figure 7). RT + IgG2a decreased DCs in brain and colon tissue compared to IgG2a alone (*p* < 0.05) and RT reduced PMN-MDSCs irrespective of immunotherapy. M2 were largely absent in peripheral blood but showed increases following brain RT + αPD-1, though this did not reach significance (Figure 7). Data show that the addition of brain or colon irradiation to αPD-1 immunotherapy may modulate in the peripheral immune response.

### Plasma Cytokines, Chemokines, and Growth Factors Were Not Altered by Combination Therapy

**Figure 8:**
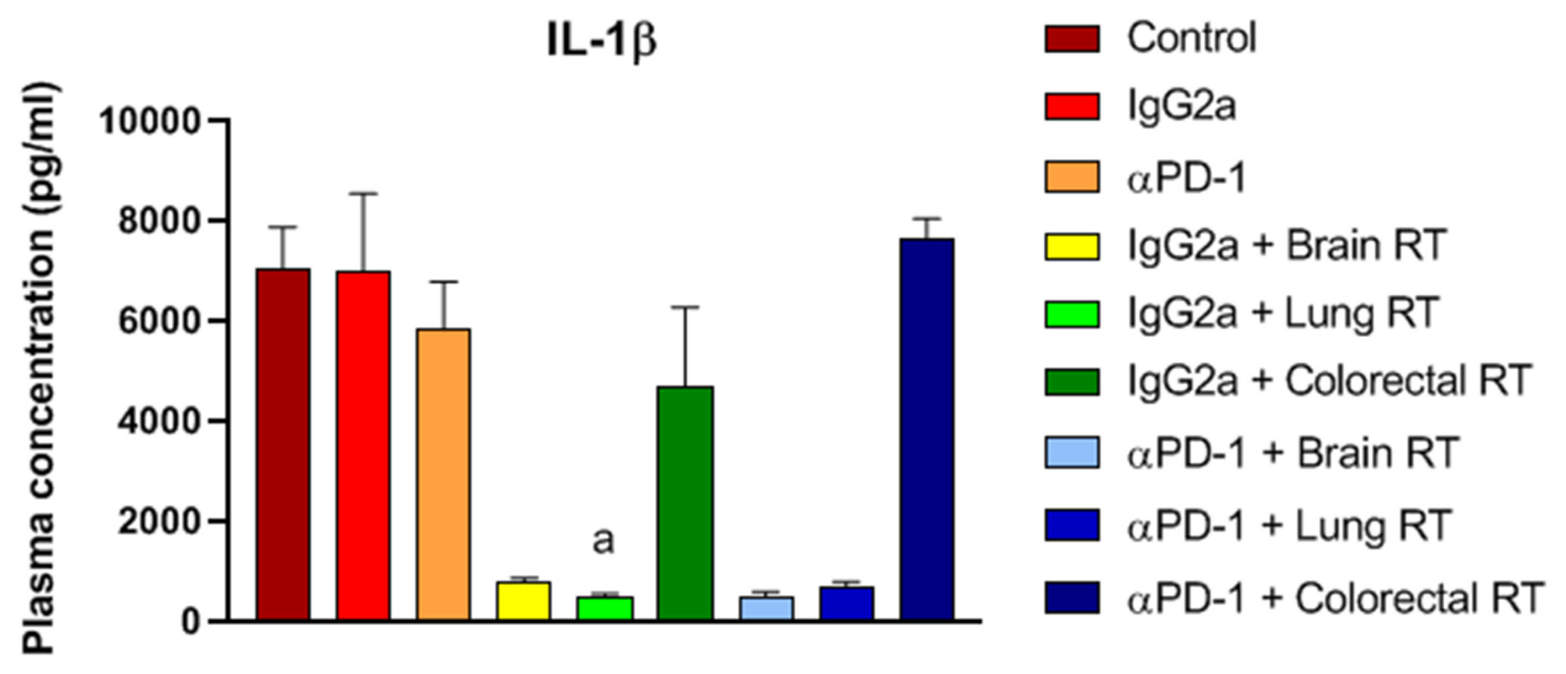
IL-1β levels from mice treated with αPD-1 and RT. Plasma was harvested at day 28 and quantified by 23-plex immunoassay (additional cytokines are presented in Supplementary Figure 6). Data are expressed as mean ± SD observed concentration in pg/ml. *N* = 6 mice per treatment group. Two Way ANOVA ^a^*p* < 0.01 vs. IgG2a by Tukey's Multiple Comparison Test.

To assess the cytokine and chemokine release following 10Gy/5 fractionated stereotactic radiation and αPD-1 immunotherapy plasma cytokine and chemokine levels were assessed by multiplex immunoassay. Irradiation of normal brain and lung tissue with or without αPD-1 decreased interleukin (IL)-1β levels 7 to 13-fold when compared to IgG2a or αPD-1 alone (Figure 8). Of note, TGF-β levels were below the level of detection in 17/48 (35%) of plasma samples (Supplementary Figure 6). Data suggest that at the sub-acute time point of 28 days post treatment commencement radiation-induced reduction of IL-1β suppression is neither ameliorated nor exacerbated by αPD-1 immunotherapy.

## Discussion

**Figure 9:**
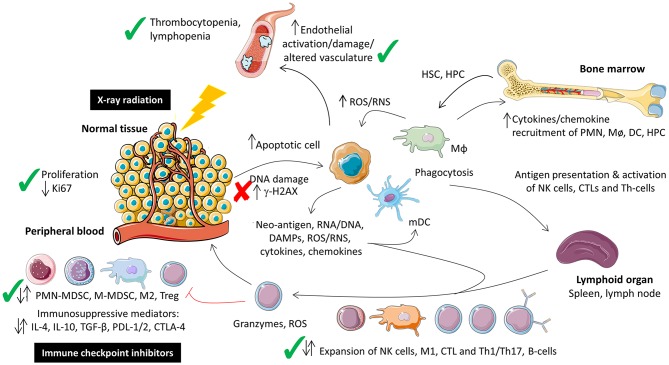
Schematic representation of the local tissue and systemic immune response to combination fractionated stereotactic radiotherapy and αPD-1 immunotherapy at day 28 post-treatment. Figure modified from McKelvey et al. (8), Creative Commons Attribution 4.0 International License (http://creativecommons.org/licenses/by/4.0/) and prepared using Sevier Medical Art (https://smart.servier.com/). Check (✓) and cross marks (✗) indicate where the data was consistent with what has been described for monotherapeutic radiotherapy and immunotherapy monotherapy. Due to the paucity of data on the combination therapy it was unclear whether immune cell populations were expected to increase, decrease or remain unchanged at this acute time point, denoted by dual arrows. CTL, cytotoxic T-lymphocyte; HPC, haematopoietic progenitor cell; HSC, haematopoetic stem cell; Mø, monocyte; MΦ, macrophage; MDSC, myeloid-derived suppressor cell.

Radiation induces DNA damage, cellular stress, apoptosis, cytokine release, and immune cell recruitment and activation (8). The effect of radiation on the tumor microenvironment is dependent on type, dose, field size, and fractionation (8). While this is known in the context of tumors, less is known regarding the systemic effect in response to local irradiation of normal tissues particularly when combined with immune-oncology agents. In this study the local tissue and systemic immune response of combined fractionated stereotaxic RT and αPD-1 immunotherapy was assessed in normal tissues that commonly (lung and colon) and infrequently (brain) experience immune-related toxicity. A schematic of the existing normal tissue response to radiation and immunotherapy, and the data summarized in this manuscript is provided in Figure 9.

The effect of combined radiation and αPD-1 immunotherapy on proliferative rates of normal tissues was assessed by Ki67^+^ staining. Ki67 is expressed during all active phases of the cell cycle (G1, S, G2, and M), but not resting cells (G0) allowing an assessment of the growth fraction of the irradiated cell populations. The mammalian intestinal epithelium rapidly renews itself, with the entire epithelium being replaced in 3–5 days. Additionally, it is known that following radiation injury, quiescent and/or radioresistant intestinal stem cells become active stem cells to regenerate the epithelium (17). This process incorporates three phases; apoptotic phase (day 1–2), proliferative phase (days 3–7) and the normalization phase (days 7–14) (17). In spite of this 2-week restorative time line and fractionated 10Gy/5 radiation treatment regimen utilized in our study, the normal colon tissue showed reduced Ki67^+^ 28 days post-irradiation of the intestinal epithelial cells. While this was not further exacerbated by the addition of αPD-1 immunotherapy, the data indicate that colon tissue is susceptible to persistent radiation-induced changes to cellular proliferation and attribute attributed to the development of colitis following radiotherapy.

The tumor vasculature and endothelial cells are some of the most studied components to assess radiobiological effects in the tumor microenvironment following radiation treatment. It is well-characterized that radiation induces endothelial cell dysfunction, including increased permeability, detachment from the underlying basement membrane, and endothelial cell senescence and/or apoptosis (18, 19). In normal brain tissue the αPD-1 immunotherapy alone increased blood vasculature while in combination with RT blood vasculature reduced. The latter was not significantly different to the number of blood vessels quantitated in IgG2a + RT brain tissue, indicating that this is consistent with the known effect of radiation on endothelial cells. A preclinical study using the same strain of mouse (C57Bl/6) to investigate cerebral permeability following 40Gy/20 fractionated radiation showed no significant difference in blood brain barrier permeability at day 30 post-irradiation; blood brain barrier permeability was not significantly increased until 90 days post-irradiation (20). Differences in the observance of alterations to blood vessel numbers and dynamics may be attributable to differences in radiation delivery and assessment methodologies. Notably, this study used whole brain irradiation and fluorescein-based intravital microscopy to assess blood permeability (which has a limitation of ~1 mm in tissue depth), while our study assessed physical blood vasculature parameters by histopathology at the isocentre of our 5 × 5 mm stereotactic irradiation focused at the caudoputamen.

Under fractionated treatment regimens (with comparatively low energy photons), radiation-induced DNA damage is principally evoked via the generation of reactive oxygen species and is mediated by H2AX (21). DNA damage to a variety of cell types in the tumor and within the surrounding healthy tissue can have a range of consequences, including microvascular endothelial cell apoptosis, crypt damage, organ failure and death (18). To investigate persistent radiation-induced DNA damage we quantified γ-H2AX staining. At the sub-acute time point γ-H2AX^+^ staining was significantly reduced by 50% in irradiated colon tissue, but not when combined with αPD-1 immunotherapy. It is unclear why γ-H2AX staining was lower in αPD-1 treated tissue when compared to control and irradiated tissues. We speculate that repair of DNA damage may have occurred, but the normal proliferative rate of cells had been impacted out to the assessed 28-day post-treatment period. This has precedence with endogenous γ-H2AX being associated with cell cycle DNA replication mediated by the DNA-dependent protein kinases/checkpoint kinase 2 pathway (22). However, γ-H2AX staining was present throughout the intestinal epithelial cells at not just the intestinal progenitor cells present at the base of intestinal crypts (as noted with Ki67 staining).

Thrombocytopenia is a hematological adverse event experienced by patients during immunotherapy treatment (23). αPD-1 administered alone suppressed platelet numbers, which normalized to control levels when radiation was added. In a descriptive observational study comprising three French pharmacovigilance databases, αPD-1 immunotherapy induced thrombocytopenia in the 0.5% of cancer patients who developed immune-related hematological adverse events (23). Surprisingly we did not observe an exacerbation of reduced platelet counts due to radiation in irradiated and animals despite bone marrow being included within our 360° Arc radiation treatment regimens.

Radiation-induced inflammasome activation and apoptosis has been noted in T cells, NK/T and monocytes with sustained caspase-1 cleavage until day 7 post-radiation (24) and is reflected in the splenic compartment (25). In our study, splenic M1 and M-MDSCs were suppressed in animals receiving combination therapy, when compared to αPD-1 monotherapy. Combined these data indicate that the addition of αPD-1 to RT significantly alters the immune repertoire of the splenic compartments. αPD-1 immunotherapy suppresses T cell function primarily by inactivating CD28 signaling (26). In the present study αPD-1 immunotherapy alone decreased Treg levels in bone marrow. Tregs play a critical role in B cell differentiation from HSCs (16) and coincided with decreased B-cells in the bone marrow compartment. Increased Th, Tc and DC cells in bone marrow were observed following combined therapy when compared to IgG2a + RT potentially indicating sequestration of pro-inflammatory immune cell types in the bone marrow. Alternatively the decreased Tregs may drive increased DCs via the PD-1-dependent bidirectional regulation of these two cell types. PD-1 is a critical homeostatic regulator for Tregs by modulating proliferation, survival and apoptosis mediated by IL-2 (27). Furthermore, the reciprocal modulation of Tregs and DC/MDSCs is dependent on chemokine CCL2 and TGF-β. PD-1 and TGF-β mediate the recruitment and bidirectional regulation of Treg cells and MDSCs (28–31) and remain elevated for up to 8 weeks post-radiation (32). While we observed an inverted Treg/DC relationship, the levels of IL-2, TGF-β and CCL2/MCP-1 were not altered in the present study.

Irradiation is known to evoke an inflammation response and is associated with increases in cytokine production. For example, irradiation of whole lung tissue with 12Gy elevates serum levels of G-CSF, IL-6, CXCL1/KC, CCL2/MCP-1, CXCL10/IP-10, and IL-1α (33) and the persistent elevation of inflammatory cytokines contributes to tissue injury and immune-related adverse events (32). In murine models of radiation-induced injury the serum cytokine levels positively correlated with irradiated tissue levels, implicating blood as a surrogate marker for tissue cytokine levels (33). The only cytokine/chemokine modulated in our sub-acute study was plasma IL-1β which decreased in animals receiving irradiation to brain and lung tissue. While IL-1β is a pluripotent cytokine and plays a role in tumorigenesis and tumor progression, the role of IL-1β in radiation-induced normal tissue toxicity is unclear (32) but has been related to skin-related adverse events (34, 35). We did not observe skin irritation from the fractionated stereotactic radiation used in our study.

Immune toxicities from radiotherapy and immunotherapy alone have been extensively reported. These include the recent establishment of European and American clinical guidelines for the management of immune toxicities which varies with grade from continuation of immunotherapy with monitoring, withholding immunotherapy and administering immunosuppressant (prednisolone), to permanent discontinuation of the immunotherapy (4, 5). What remains comparatively unknown is whether combining radiation and immune check point immunotherapy will exacerbate these immune-related toxicities and whether these can be predicted at early time points during the treatment regimen. Overall our acute snapshot of this dynamic response showed that blood vasculature, cell proliferation, thrombocytopenia, lymphopenia, immune suppression and altered immune repertoire (including IL-1β) are observed when combination therapy of fractionated stereotactic radiotherapy and αPD-1 immunotherapy was used compared to either monotherapy. Consistent with low number of clinical studies on concurrent or sequential radiotherapy and immunotherapy there were no increases in serious acute toxicity from the combination therapy in our preclinical model when compared to monotherapy (36), but longer term studies are required. Akin to the clinical data, our report supports close monitoring of immune-related adverse events in patients who are to receive combination therapy. IL-1β and peripheral blood M2 could be further explored as potential biomarkers for immune toxicity.

## Data Availability Statement

The datasets generated for this study are available on request to the corresponding author.

## Ethics Statement

The animal study was reviewed and approved by Northern Sydney Local Heath District Animal Ethics Committee, Royal North Shore Hospital, St Leonards, Australia (Approval #RESP/17/205).

## Author Contributions

KM, TE, SC, HW, CD, and VH contributed conception and design of the study. KM performed the animal experimentation, histopathology, hematology, flow cytometry, performed the statistical analyses, and wrote the first draft of the manuscript. RP contributed to the histopathology. AH performed the multiplex immunoassay. All authors contributed to manuscript revision, read, and approved the submitted version.

### Conflict of Interest

The authors declare that the research was conducted in the absence of any commercial or financial relationships that could be construed as a potential conflict of interest.

## References

[1] Carbone DP Reck M Paz-Ares L Creelan B Horn L Steins M First-line nivolumab in stage IV or recurrent non-small-cell lung cancer. N Engl J Med. (2017) 376:2415–26. 10.1056/NEJMoa1613493 28636851 PMC6487310

[2] Mantia CM Buchbinder EI. Immunotherapy toxicity. Hematol Oncol Clin N Am. (2019) 33:275–90. 10.1016/j.hoc.2018.12.008 30833000

[3] Puzanov I Diab A Abdallah K Bingham CO III Brogdon C Dadu R. Managing toxicities associated with immune checkpoint inhibitors: consensus recommendations from the Society for Immunotherapy of Cancer (SITC) Toxicity Management Working Group. J Immunother Cancer. (2017) 5:95. 10.1186/s40425-017-0300-z 29162153 PMC5697162

[4] Haanen J Carbonnel F Robert C Kerr KM Peters S Larkin J. Management of toxicities from immunotherapy: ESMO Clinical Practice Guidelines for diagnosis, treatment and follow-up. Ann Oncol. (2018) 29(Suppl 4):iv264–6. 10.1093/annonc/mdy162 29917046

[5] Brahmer JR Lacchetti C Schneider BJ Atkins MB Brassil KJ Caterino JM. Management of immune-related adverse events in patients treated with immune checkpoint inhibitor therapy: american society of clinical oncology clinical practice guideline. J Clin Oncol. (2018) 36:1714–68. 10.1200/JCO.2017.77.6385 29442540 PMC6481621

[6] Baxi S Yang A Gennarelli RL Khan N Wang Z Boyce L. Immune-related adverse events for anti-PD-1 and anti-PD-L1 drugs: systematic review and meta-analysis. BMJ. (2018) 360:k793. 10.1136/bmj.k793 29540345 PMC5851471

[7] Cortazar FB Marrone KA Troxell ML Ralto KM Hoenig MP Brahmer JR. Clinicopathological features of acute kidney injury associated with immune checkpoint inhibitors. Kidney Int. (2016) 90:638–47. 10.1016/j.kint.2016.04.008 27282937 PMC4983464

[8] McKelvey KJ Hudson AL Back M Eade T Diakos CI. Radiation, inflammation and the immune response in cancer. Mammal Genome. (2018) 29:843–65. 10.1007/s00335-018-9777-0 30178305 PMC6267675

[9] Qi ZY Deng XW Huang SM Lu J Lerch M Cutajar D. Verification of the plan dosimetry for high dose rate brachytherapy using metal-oxide-semiconductor field effect transistor detectors. Med Phys. (2007) 34:2007–13. 10.1118/1.2736288 17654904

[10] Kwan IS Rosenfeld AB Qi ZY Wilkinson D Lerch MLF Cutajar DL Skin dosimetry with new MOSFET detectors. Radiat Meas. (2008) 43:929–32. 10.1016/j.radmeas.2007.12.052

[11] Breitkreutz DY Bialek S Vojnovic B Kavanagh A Johnstone CD Rovner Z. A 3D printed modular phantom for quality assurance of image-guided small animal irradiators: design, imaging experiments, and Monte Carlo simulations. Med Phys. (2019) 46:2015–24. 10.1002/mp.13525 30947359

[12] Dogdas B Stout D Chatziioannou AF Leahy RM. Digimouse: a 3D whole body mouse atlas from CT and cryosection data. Phys Med Biol. (2007) 52:577–87. 10.1088/0031-9155/52/3/003 17228106 PMC3006167

[13] Stout D Chow P Silverman R Leahy RM Lewis X Gambhir S Creating a whole body digital mouse atlas with PET, CT and cryosection images. Mol Imaging Biol. (2002) 4:S27 Available online at: https://neuroimage.usc.edu/neuro/Digimouse

[14] Reyes-Aldasoro CC Williams LJ Akerman S Kanthou C Tozer GM. An automatic algorithm for the segmentation and morphological analysis of microvessels in immunostained histological tumour sections. J Microscopy. (2011) 242:262–78. 10.1111/j.1365-2818.2010.03464.x 21118252

[15] Kuo LJ Yang LX. γ-H2AX - a novel biomarker for DNA double-strand breaks. In vivo. (2008) 22:305–9. Available online at: http://iv.iiarjournals.org/content/22/3/305.long 18610740

[16] Rajani KR Carlstrom LP Parney IF Johnson AJ Warrington AE Burns TC. Harnessing radiation biology to augment immunotherapy for glioblastoma. Front Oncol. (2019) 8:656. 10.3389/fonc.2018.00656 30854331 PMC6395389

[17] Kim C-K Yang VW Bialkowska AB. The role of intestinal stem cells in epithelial regeneration following radiation-induced gut injury. Curr Stem Cell Rep. (2017) 3:320–32. 10.1007/s40778-017-0103-7 29497599 PMC5818549

[18] Paris F Fuks Z Kang A Capodieci P Juan G Ehleiter D. Endothelial apoptosis as the primary lesion initiating intestinal radiation damage in mice. Science. (2001) 293:293. 10.1126/science.1060191 11452123

[19] Venkatesulu BP Mahadevan LS Aliru ML Yang X Bodd MH Singh PK. Radiation-induced endothelial vascular injury: a review of possible mechanisms. JACC Basic Transl Sci. (2018) 3:563–72. 10.1016/j.jacbts.2018.01.014 30175280 PMC6115704

[20] Yuan H Gaber MW Boyd K Wilson CM Kiani MF Merchant TE. Effects of fractionated radiation on the brain vasculature in a murine model: blood-brain barrier permeability, astrocyte proliferation, and ultrastructural changes. Int J Radiat Oncol Biol Phys. (2006) 66:860–6. 10.1016/j.ijrobp.2006.06.043 17011458

[21] Kang MA So EY Simons AL Spitz DR Ouchi T. DNA damage induces reactive oxygen species generation through the H2AX-Nox1/Rac1 pathway. Cell Death Dis. (2012) 3:e249. 10.1038/cddis.2011.134 22237206 PMC3270268

[22] Tu W-Z Li B Huang B Wang Y Liu X-D Guan H. γH2AX foci formation in the absence of DNA damage: mitotic H2AX phosphorylation is mediated by the DNA-PKcs/CHK2 pathway. FEBS Lett. (2013) 587:3437–43. 10.1016/j.febslet.2013.08.028 24021642

[23] Delanoy N Michot JM Comont T Kramkimel N Lazarovici J Dupont R. Haematological immune-related adverse events induced by anti-PD-1 or anti-PD-L1 immunotherapy: a descriptive observational study. Lancet Haematol. (2019) 6:e48–57. 10.1016/S2352-3026(18)30175-3 30528137

[24] Stoecklein VM Osuka A Ishikawa S Lederer MR Wanke-Jellinek L Lederer JA. Radiation exposure induces inflammasome pathway activation in immune cells. J Immunol. (2015) 194:1178–89. 10.4049/jimmunol.1303051 25539818 PMC4326002

[25] Balogh A Persa E Bogdándi EN Benedek A Hegyesi H Sáfrány G. The effect of ionizing radiation on the homeostasis and functional integrity of murine splenic regulatory T cells. Inflammation Res. (2013) 62:201–12. 10.1007/s00011-012-0567-y 23080082

[26] Hui E Cheung J Zhu J Su X Taylor MJ Wallweber HA. T cell costimulatory receptor CD28 is a primary target for PD-1-mediated inhibition. Science. (2017) 355:1428–33. 10.1126/science.aaf1292 28280247 PMC6286077

[27] Asano T Meguri Y Yoshioka T Kishi Y Iwamoto M Nakamura M. PD-1 modulates regulatory T-cell homeostasis during low-dose interleukin-2 therapy. Blood. (2017) 129:2186–97. 10.1182/blood-2016-09-741629 28151427 PMC5391624

[28] Lee CR Kwak Y Yang T Han JH Park SH Ye MB. Myeloid-derived suppressor cells are controlled by regulatory T cells via TGF-beta during murine colitis. Cell Rep. (2016) 17:3219–32. 10.1016/j.celrep.2016.11.062 28009291

[29] Zhang Y Velez-Delgado A Mathew E Li D Mendez FM Flannagan K. Myeloid cells are required for PD-1/PD-L1 checkpoint activation and the establishment of an immunosuppressive environment in pancreatic cancer. Gut. (2017) 66:124–36. 10.1136/gutjnl-2016-312078 27402485 PMC5256390

[30] Shurin GV Ma Y Shurin MR. Immunosuppressive mechanisms of regulatory dendritic cells in cancer. Cancer Microenviron. (2013) 6:159–67. 10.1007/s12307-013-0133-3 23749739 PMC3717058

[31] Chang AL Miska J Wainwright DA Dey M Rivetta CV Yu D. CCL2 produced by the glioma microenvironment is essential for the recruitment of regulatory T cells and myeloid-derived suppressor cells. Cancer Res. (2016) 76:5671–82. 10.1158/0008-5472.CAN-16-0144 27530322 PMC5050119

[32] Rubin P Johnston CJ Williams JP McDonald S Finkelstein JN. A perpetual cascade of cytokines postirradiation leads to pulmonary fibrosis. Int J Radiat Oncol Biol Phys. (1995) 33:99–109. 10.1016/0360-3016(95)00095-G 7642437

[33] Ao X Zhao L Davis MA Lubman DM Lawrence TS Kong FM. Radiation produces differential changes in cytokine profiles in radiation lung fibrosis sensitive and resistant mice. J Hematol Oncol. (2009) 2:6. 10.1186/1756-8722-2-6 19187543 PMC2663566

[34] Liu W Ding I Chen K Olschowka J Xu J Hu D. Interleukin 1beta (IL1B) signaling is a critical component of radiation-induced skin fibrosis. Radiat Res. (2006) 165:181–91. 10.1667/RR3478.1 16435917

[35] Janko M Ontiveros F Fitzgerald TJ Deng A DeCicco M Rock KL. IL-1 generated subsequent to radiation-induced tissue injury contributes to the pathogenesis of radiodermatitis. Radiat Res. (2012) 178:166–72. 10.1667/RR3097.1 22856653 PMC3483593

[36] Verma V Cushman TR Tang C Welsh JW. Toxicity of radiation and immunotherapy combinations. Adv Radiat Oncol. (2018) 3:506–11. 10.1016/j.adro.2018.08.003 30370349 PMC6200891

