# Supplementary material for: Sub-acute Toxicity in Non-cancerous Tissue and Immune-Related Adverse Events of a Novel Combination Therapy for Cancer
Source: Front Oncol. 2020 Jan 14;9:1504. doi: 10.3389/fonc.2019.01504 (PMC6971197; doi:10.3389/fonc.2019.01504)
Supplement: Supplementary file 1 [file Table_1.DOCX]

**Supplementary Table I.** Mean radiation dose to targeted tissue and organs at risk

| **Structure** | **Volume (cc)** | **Mean dose (cGy)** | **Min dose (cGy)** | **Max dose (cGy)** |
| --- | --- | --- | --- | --- |
| *Brain* |  |  |  |  |
| Targeted tissue | 0.02 | 199.11 | 184.18 | 204.73 |
| Brain | 0.41 | 84.88 | 1.47 | 310.93 |
| Spinal cord | 0.13 | 0.71 | 0.00 | 3.42 |
| Inner ear | 0.01 | 21.68 | 0.00 | 237.63 |
| Eyes | 0.11 | 0.39 | 0.00 | 3.17 |
| *Lung* |  |  |  |  |
| Targeted tissue | 0.06 | 169.57 | 3.67 | 318.38 |
| Right lung | 0.57 | 26.55 | 0.00 | 201.76 |
| Left lung | 0.33 | 9.38 | 0.00 | 68.64 |
| Heart | 0.31 | 5.69 | 0.00 | 51.3 |
| Spinal cord | 0.13 | 1.50 | 0.04 | 58.21 |
| *Colon* |  |  |  |  |
| Targeted tissue | 0.06 | 169.57 | 3.67 | 318.38 |
| Caecum | 0.14 | 0.19 | 0.00 | 0.54 |
| Small intestine | 1.15 | 0.76 | 0.00 | 285.80 |
| Anorectum | 0.10 | 60.95 | 0.23 | 319.80 |
| Bladder | 0.06 | 0.69 | 0.18 | 1.16 |
| Stomach | 0.16 | 0.07 | 0.00 | 0.31 |
| Spinal cord | 0.13 | 0.08 | 0.01 | 1.25 |

Representative dose and volume parameters determined by tissue contouring in MuriPlan Treatment Planning System. cc, cubic centimetre; cGy, centi-Gray = 0.1 Gray.
