# Supplementary material for: Sub-acute Toxicity in Non-cancerous Tissue and Immune-Related Adverse Events of a Novel Combination Therapy for Cancer
Source: Front Oncol. 2020 Jan 14;9:1504. doi: 10.3389/fonc.2019.01504 (PMC6971197; doi:10.3389/fonc.2019.01504)
Supplement: Supplementary file 2 [file Table_2.DOCX]

**Supplementary Figure 1.** Radiation treatment planning and representative dose volume histogram. A single 360^o^ (^-^180^o^ to 180^o^) sagittal arc was planned using the 5×5 collimator and representative dose volume histogram of the planning treatment volume, contoured target tissue and organs at risk.

**Supplementary Figure 2.** Animal weights following treatment with αPD-1 or IgG2a isotype control, with or without irradiation to the brain, colon or lung region. Symbols denote mean percentage (%) normalized to baseline weight ± SD weight.

**Supplementary Figure 3.** Systemic immune cell populations from mice treated with αPD-1 and/or 10Gy/5 X-ray irradiation of brain tissue. Cells were harvested at day 28 and quantitated by multi-color flow cytometry. Data are expressed as mean ± SD percentage of parent population (%; N=6 mice per treatment group). Two Way ANOVA ^a^p<0.05 vs IgG2a; ^b^p<0.05 vs αPD-1; ^c^p<0.05 vs IgG2a + RT by Tukey’s Multiple Comparison Test.

**Supplementary Figure 4.** Systemic immune cell populations from mice treated with αPD-1 and/or 10Gy/5 X-ray irradiation of lung tissue. Cells were harvested at day 28 and quantitated by multi-color flow cytometry. Data are expressed as mean ± SD percentage of parent population (%; N=6 mice per treatment group). Two Way ANOVA ^a^p<0.05 vs IgG2a; ^b^p<0.05 vs αPD-1; ^c^p<0.05 vs IgG2a + RT by Tukey’s Multiple Comparison Test.

**Supplementary Figure 5.** Systemic immune cell populations from mice treated with αPD-1 and/or 10Gy/5 X-ray irradiation of colon tissue. Cells were harvested at day 28 and quantitated by multi-color flow cytometry. Data are expressed as mean ± SD percentage of parent population (%; N=6 mice per treatment group). Two Way ANOVA ^a^p<0.05 vs IgG2a; ^b^p<0.05 vs αPD-1; ^c^p<0.05 vs IgG2a + RT by Tukey’s Multiple Comparison Test.

**Supplementary Figure 6.** Plasma cytokine and chemokines levels from mice treated with αPD-1 and RT. Plasma was harvested at day 28 and quantified by 23-plex immunoassay and sandwich ELISA (TGF-β1). Data are expressed as mean ± SD observed concentration in pg/ml. N=6 mice per treatment group. Two Way ANOVA ^a^p<0.01 vs IgG2a by Tukey’s Multiple Comparison Test.
