# Supplementary figures and images for: Sub-acute Toxicity in Non-cancerous Tissue and Immune-Related Adverse Events of a Novel Combination Therapy for Cancer
Source: Front Oncol. 2020 Jan 14;9:1504. doi: 10.3389/fonc.2019.01504 (PMC6971197; doi:10.3389/fonc.2019.01504)

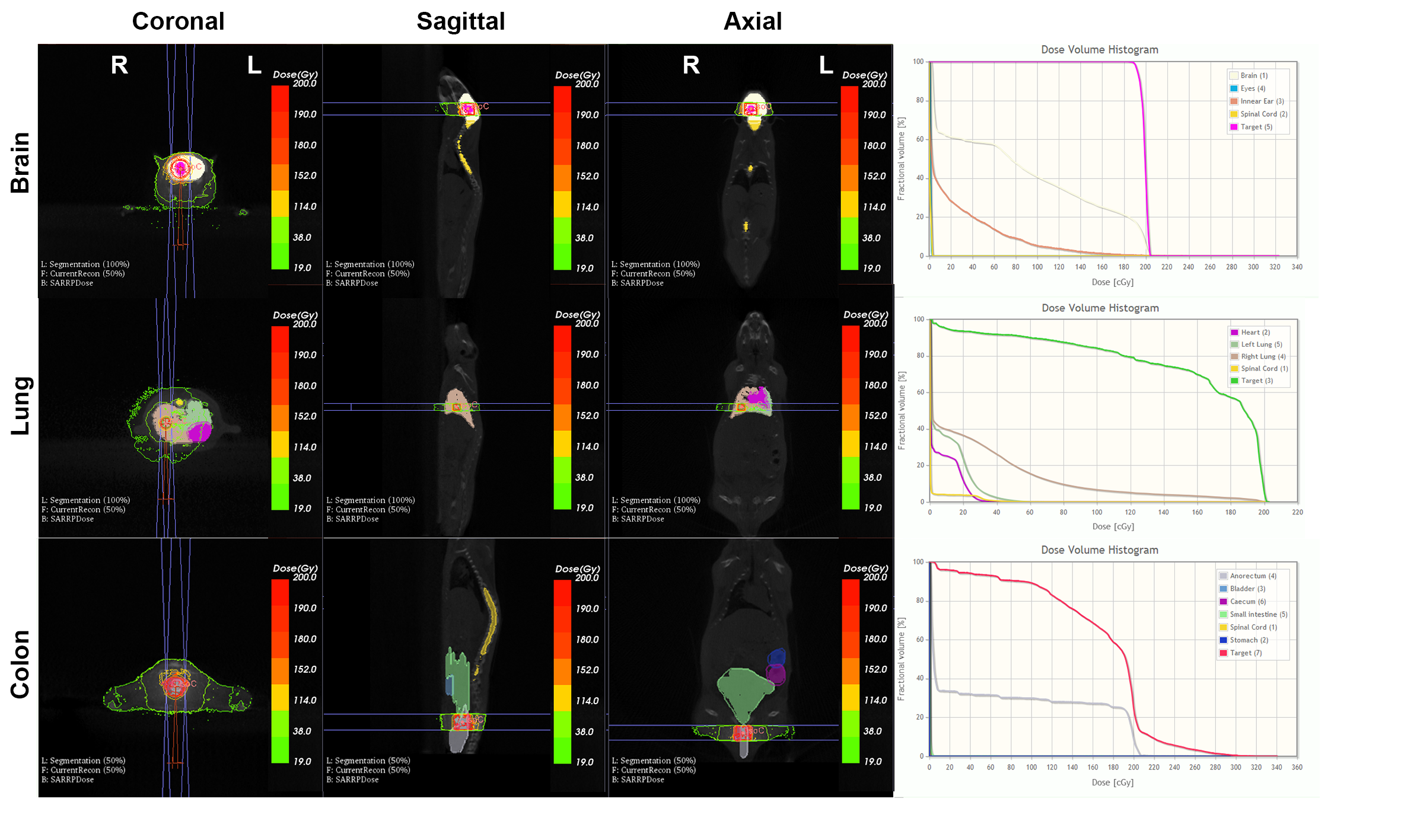

Supplement: Supplementary file 3 [file Image_1.TIF]

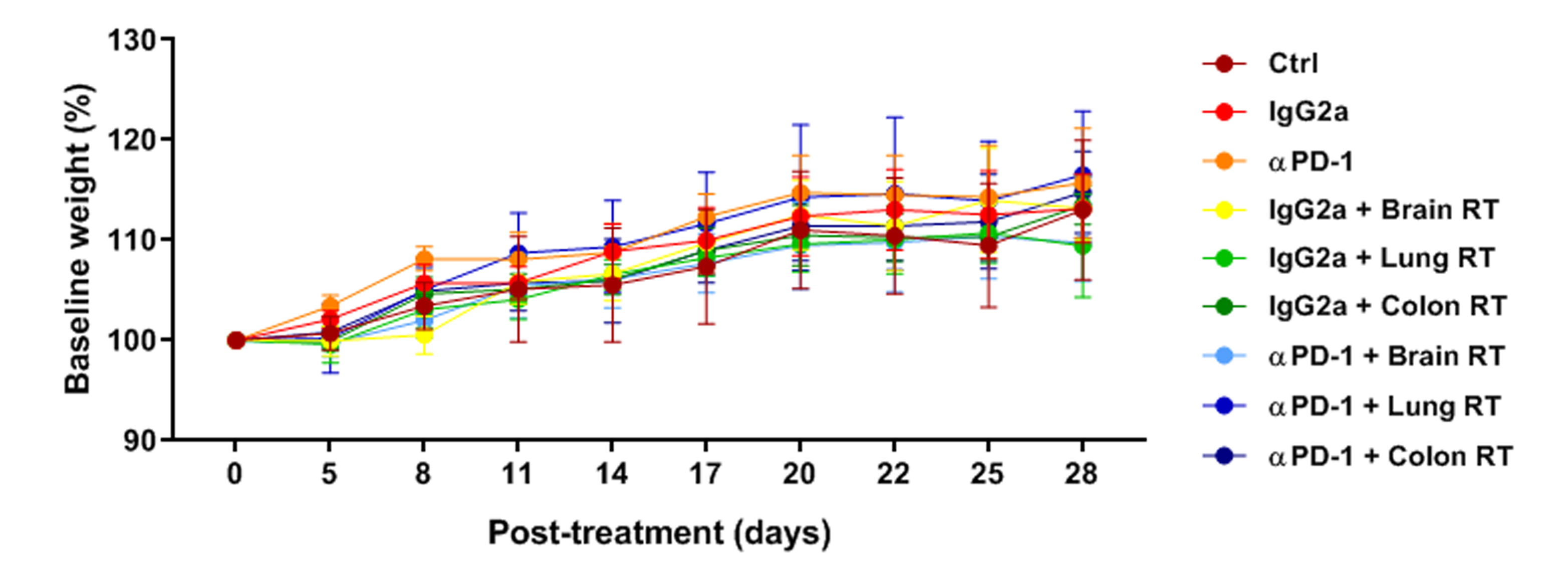

Supplement: Supplementary file 4 [file Image_2.TIF]

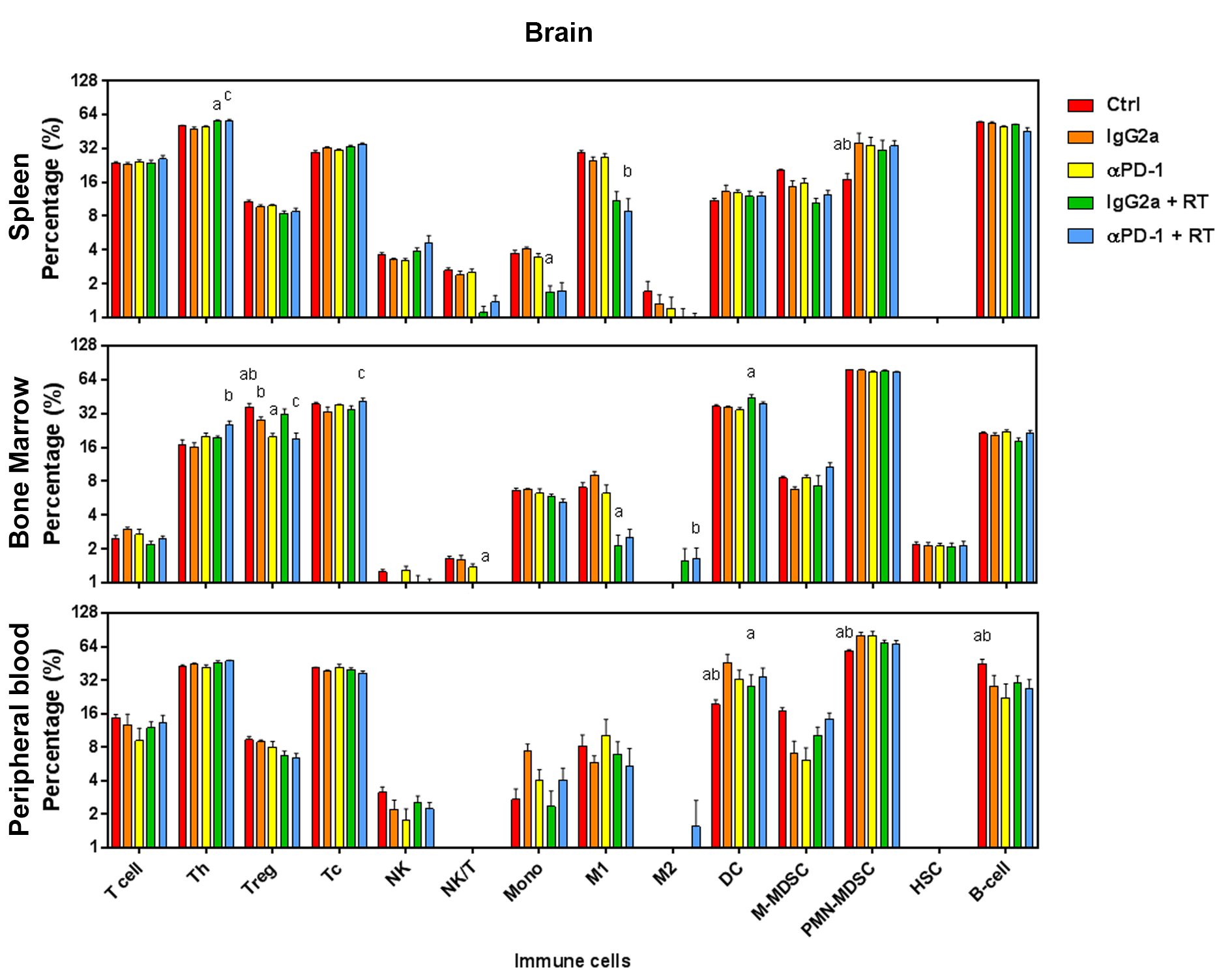

Supplement: Supplementary file 5 [file Image_3.TIF]

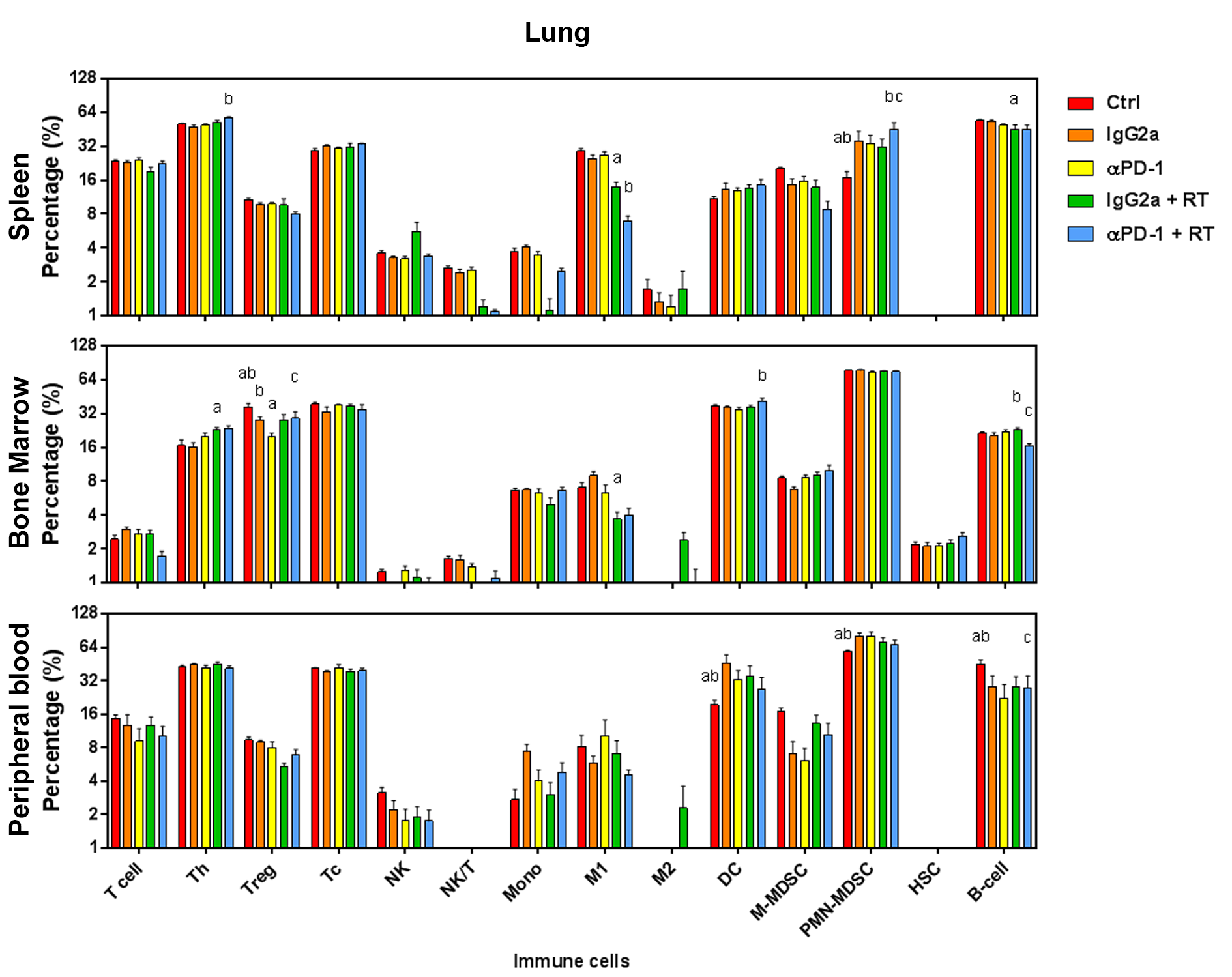

Supplement: Supplementary file 6 [file Image_4.TIF]

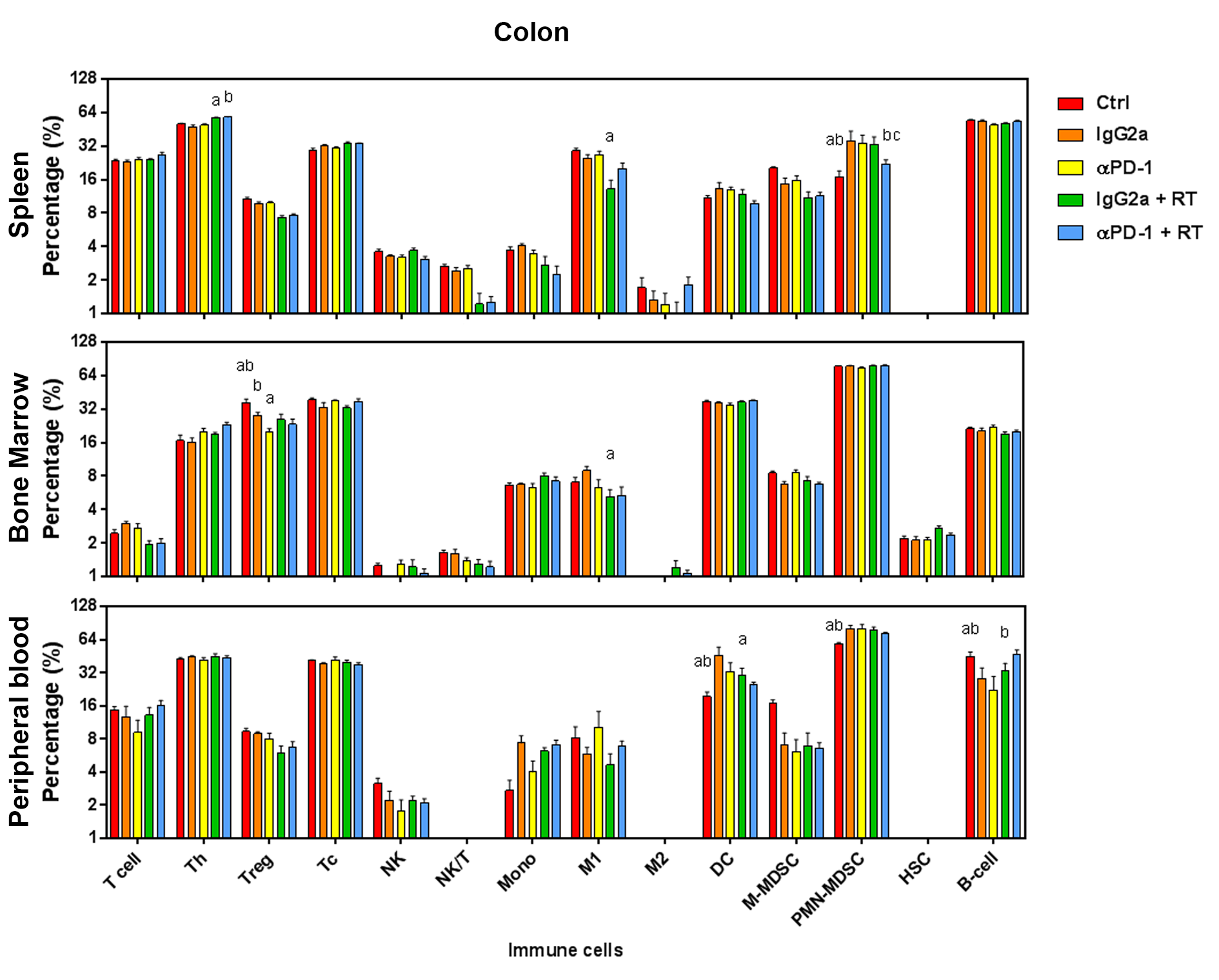

Supplement: Supplementary file 7 [file Image_5.TIF]

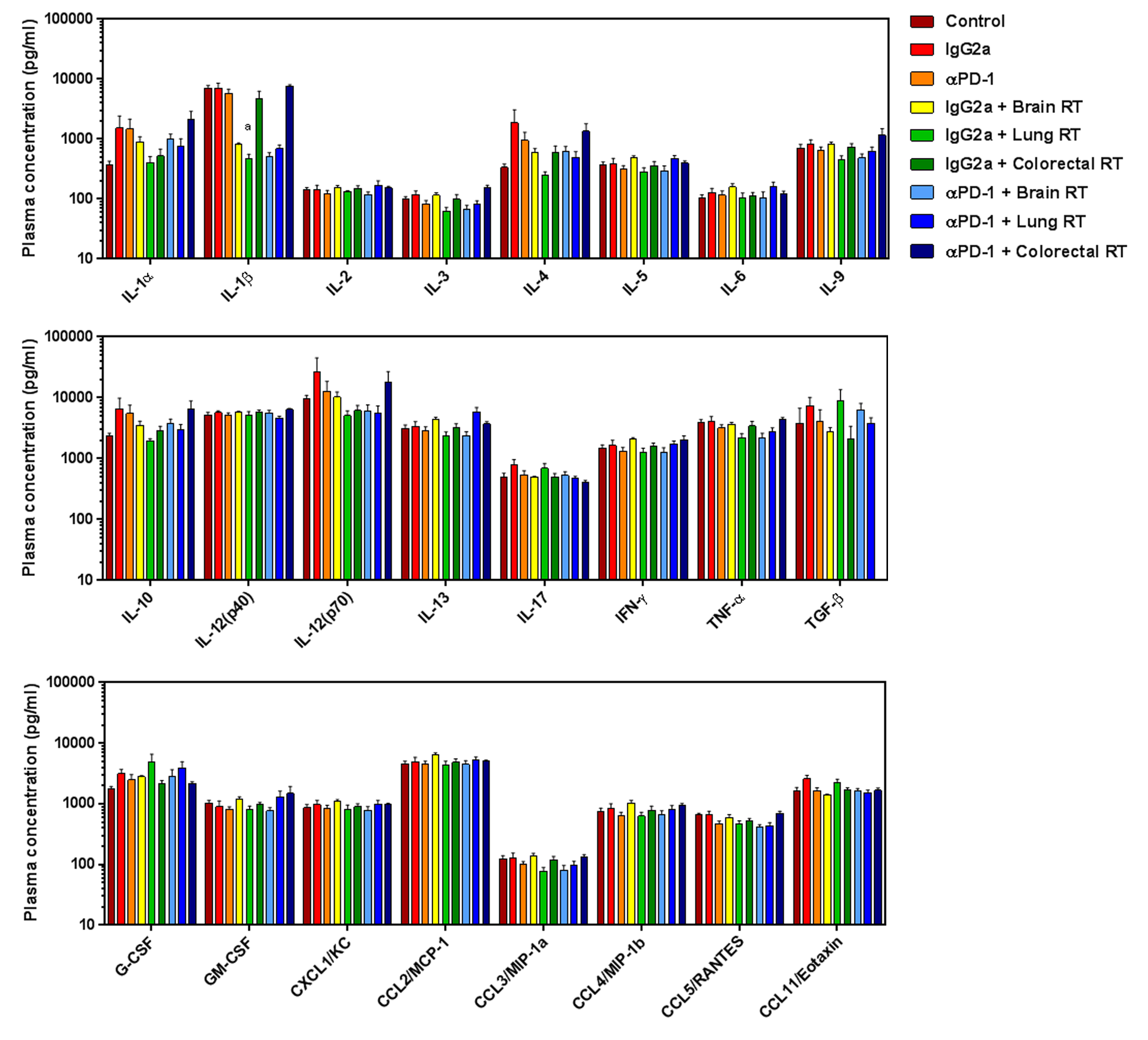

Supplement: Supplementary file 8 [file Image_6.TIF]
